# Rationale for using lenvatinib as an urgent initial therapy in thyroid cancer with remarkable laryngotracheal invasion

**DOI:** 10.1530/ETJ-25-0337

**Published:** 2025-12-24

**Authors:** Emmanuel Martinod, Paulina Kuczma, Christophe Trésallet, Cécile Ghander, Camille Buffet, Eric Vicaut

**Affiliations:** ^1^ Assistance Publique – Hôpitaux de Paris (AP-HP), Hôpitaux Universitaires Paris Seine-Saint-Denis, Hôpital Avicenne, Chirurgie Thoracique et Vasculaire, Université Sorbonne Paris Nord, Faculté de Médecine SMBH, Bobigny, France; ^2^ Inserm UMR1272, Hypoxie et Poumon, Université Sorbonne Paris Nord, Faculté de Médecine SMBH, Bobigny, France; ^3^ Université Paris Cité, Fondation Alain Carpentier, Laboratoire de Recherche Bio-chirurgicale, AP-HP, Hôpital Européen Georges Pompidou, Paris, France; ^4^ AP-HP, Hôpitaux Universitaires Paris Seine-Saint-Denis, Hôpital Avicenne, Chirurgie Digestive et Endocrinienne, Université Sorbonne Paris Nord, Faculté de Médecine SMBH, Bobigny, France; ^5^ AP-HP Sorbonne Université, Hôpital La Pitié-Salpêtrière, Service des Pathologies Thyroïdiennes et Tumorales Endocrines, Paris, France; ^6^ AP-HP, Unité de Recherche Clinique, Hôpitaux Saint Louis-Lariboisière-Fernand Widal, Université Paris Cité – all in France, Paris, France

**Keywords:** thyroid cancer, trachea, larynx, lenvatinib, tracheal replacement

Dear Editor,

We read with great interest the article entitled *‘*Lenvatinib as an urgent initial therapy in thyroid cancer with remarkable laryngotracheal invasion*’,* published in the *European Thyroid Journal* by Katoh and colleagues (1). The study evaluated the use of lenvatinib (LEN) as an initial and bridging treatment for patients with advanced thyroid cancer causing severe airway obstruction. In the 14 patients with significant laryngotracheal invasion, LEN rapidly reduced tumor size and improved airway diameter in nearly all cases, relieving symptoms within a few days. Only one patient developed a controlled fistula. The findings suggest that LEN provides a fast, noninvasive alternative to emergency airway procedures and serves as an effective bridging therapy during genetic testing in anaplastic thyroid cancer (ATC).

In the introduction, we read the following: ‘Remarkable laryngotracheal invasion can cause severe airway stenosis, requiring urgent treatment including radical resection, tracheostomy, and so forth. Undoubtedly the initial treatment of locally advanced differentiated thyroid cancer (DTC) is radical resection, however, which may need to sacrifice the critical structures in order to achieve complete curative resection…In such life-threatening cases with laryngotracheal invasion of thyroid cancer, the urgent curative surgery combined with tracheal resection or laryngectomy, tracheostomy, palliative stenting, or laser bronchoscopy would be treatment options’. It is surprising that at no point, particularly in the introduction or discussion, tracheal or laryngotracheal replacement is considered in the context of extensive DTC. A recent systematic review indicated that tracheal replacement was primarily performed for malignant lesions (2). The most frequent causes were thyroid cancers invading the trachea and adenoid cystic carcinomas, followed by lung carcinoma, squamous cell carcinoma, and mucoepidermoid carcinoma. In cases of DTC with tracheal invasion, prognosis depends on achieving complete surgical resection, as other treatments are not curative. Radical surgery, combined with tracheal replacement when necessary, plays a crucial role in managing locally invasive T4 tumors and improving long-term outcomes. The TRITON-01 registry, together with a pioneering prospective study, showed that 12 patients with advanced thyroid or parathyroid cancer underwent airway reconstruction using stented aortic matrices between 2016 and 2023 (3, 4, 5). All patients had cricotracheal resection with cryopreserved allograft reconstruction, achieving complete (R0) resection with no intraoperative complications or perioperative deaths. Postoperative issues, such as airway infection or granuloma formation, were manageable. Over an average follow-up of 3 years, all ten surviving patients were breathing and speaking normally, with no tracheal recurrence; most showed disease remission or stability. The procedure proved to be a safe and effective alternative for previously unresectable cricotracheal invasion, avoiding common complications of traditional tracheal resection. Remarkably, pseudocartilage developed within the aortic grafts, suggesting *in vivo* airway tissue regeneration. Despite its small sample size and retrospective design, the study supports tracheal replacement with stented aortic matrices as a promising surgical option to be further evaluated in the upcoming TRITON-02 trial.

The authors concluded as follows: ‘…initial LEN therapy exhibited rapid improvement of airway obstructive symptoms in patients with marked laryngotracheal invasion, allowing to avoid urgent invasive and traumatic procedure. Bridging LEN therapy would provide significant impact particularly on ATC patients with severe airway stenosis by tumor invasion during turn-around-time (TAT) of genetic testing. Therefore, initial LEN therapy would be considered for patients with remarkable laryngotracheal invasion under careful monitoring of fistula formation’. In fact, the series included 10/14 ATCs, 1/14 medullary thyroid cancer, 2/14 poorly DTCs, and only one papillary thyroid cancer. The authors suggested that the best target for the proposed treatment is ATC, which, in our view, should have been the sole focus of this article (10 out of 14 patients). Unless we are mistaken, it appears that most patients did not undergo surgery after the treatment, which further supports that the study group should have consisted solely of ATC cases. No long-term follow-up data are available beyond the acute phase of the treatment’s effect.

Finally, the prescription of LEN for ATC has not been endorsed by either the American Thyroid Association (ATA) guidelines or the French TUTHYREF network (6, 7). The rationale for this article’s discussion of LEN in unresectable ATC appears weak, as it relies primarily on a single study suggesting some efficacy, which was not confirmed by the International Thyroid Oncology Group (ITOG) study. Moreover, the study published in the *European Thyroid Journal* considers LEN as a bridging therapy while awaiting molecular biology results, which, as the authors themselves note, can be obtained in expert centers within 7 days or even within 24 h when using digital PCR on a fine-needle aspiration sample, as performed at our center. In clinical practice, this leaves a very limited window for LEN use. These considerations call for great caution before proposing LEN as a first-line treatment option for locally advanced ATC.

## Declaration of interest

The authors declare that there is no conflict of interest that could be perceived as prejudicing the impartiality of the work reported.

## Funding

This work did not receive any specific grant from any funding agency in the public, commercial, or not-for-profit sector.
